# A Straightforward Algorithm for Diagnosing Hymenoptera Venom Allergy and Identifying the Relevant Venom for Immunotherapy

**DOI:** 10.1111/all.70154

**Published:** 2025-11-12

**Authors:** Lisa Arzt‐Gradwohl, Eva Schadelbauer, Urban Čerpes, Gudrun Pregartner, Sereina Annik Herzog, Elisabeth Jungwirth, Karin Laipold, Barbara Binder, Gunter J. Sturm

**Affiliations:** ^1^ Department of Dermatology and Venereology Medical University of Graz Graz Austria; ^2^ Institute for Medical Informatics, Statistics and Documentation Medical University of Graz Graz Austria; ^3^ Allergy Outpatient Clinic Reumannplatz Vienna Austria

**Keywords:** basophil activation test, diagnosis, hymenoptera venom allergy, intradermal test, specific IgE

## Abstract

**Background:**

Routine allergy diagnostic assessment relies on the patient's medical history, skin testing, and the detection of specific IgE (sIgE) in the serum. In addition, molecular allergy diagnostics and the basophil activation test (BAT) can be used; however, these additional tests are costly and sometimes time‐consuming. Therefore, our objective was to identify the test with the highest sensitivity, determine the most effective combination of tests to enhance sensitivity, and develop a simple IgE ratio to distinguish between bee and vespid venom allergy in cases of double positivity.

**Methods:**

We retrospectively analyzed data from 190 bee venom‐allergic and 809 vespid venom‐allergic patients, along with 61 non‐allergic subjects. Sensitivity and specificity were assessed for sIgE (ImmunoCAP), the intradermal test (IDT), and the BAT.

**Results:**

Applying a lower cut‐off of 0.1 kU/L in patients with total IgE < 30 kU/L (while keeping the standard cut‐off of 0.35 kU/L otherwise) increased sensitivity for sIgE to bee venom from 85.7% to 94.6% and for vespid venom from 92.2% to 97.0%. The highest sensitivity (100%) was achieved by combining this sIgE dual cut‐off approach with the intradermal test for bee venom allergy, while sensitivity for vespid venom allergy reached 98.5%. A vespid/bee ratio ≥ 2.68 indicated vespid venom allergy with 59.3% sensitivity, while a bee/vespid ratio ≥ 5.33 identified bee venom allergy with 50.6% sensitivity, both with 100% specificity.

**Conclusions:**

Conventional tests, such as sIgE determination or intradermal testing, are usually sufficient. Combining both increases sensitivity, while additional tests provide no further benefit. Double‐positive findings may often be clarified using a simple sIgE ratio, though sensitivity remains limited.

## Graphical abstract

Conventional tests, such as specific IgE (sIgE) or intradermal testing (IDT), are usually sufficient. Combining both tests increases sensitivity, while additional tests, like the basophil activation test (BAT), provide no further benefit for the majority of patients. The simple sIgE ratio allows quick exclusion of irrelevant double sensitizations without the need for additional expensive tests. BAT, basophil activation test; BV, bee venom; IDT, intradermal test; sIgE, specific IgE; VV, vespid venom.

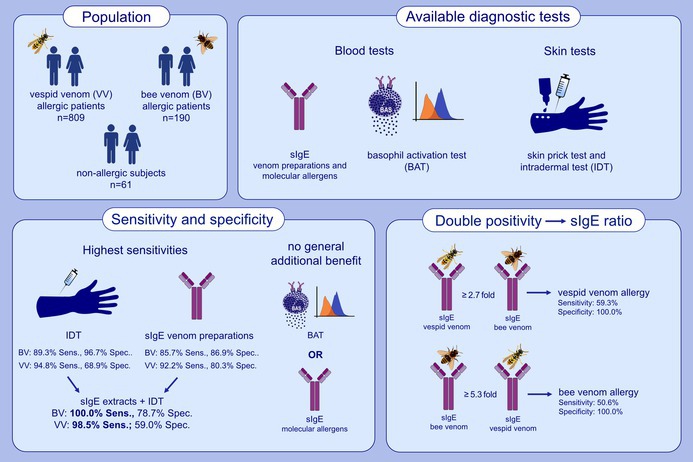

**Abbreviations**
BAT: basophil activation test
CAP‐FEIA: carrier polymer system—fluorescent enzyme immunoassay
CCD: cross‐reactive carbohydrate determinant
HVA: hymenoptera venom allergy
IDT: intradermal test
MAD: molecular allergy diagnosis
MUXF: mannosylated xylose‐containing fucosylated glycan
RAST: radio‐allergo‐sorbent test
sIgE: specific immunoglobulin E
SPT: skin prick test
tIgE: total immunoglobulin E
VIT: venom immunotherapy

## 1 Background

Hymenoptera stings are common, with 56.6%–94.5% of the general population reporting having been stung at least once in their lifetime [1]. Moreover, venom hypersensitivity is the leading cause of anaphylactic reactions in adults across Europe [2]. In European epidemiological studies, the prevalence of self‐reported systemic sting reactions in adults ranges from 0.3% to 7.5% [3].

In general, there are two major problems in the diagnosis of Hymenoptera venom allergy (HVA). First, there is low specificity at certain test concentrations, which may lead to unnecessary venom immunotherapy. Second, insufficient sensitivity can lead to false‐negative results and thereby increase the risk of subsequent systemic sting reactions; this limitation currently applies in particular to molecular allergy diagnostics.

The routine diagnosis of HVA relies on the patient's medical history, skin testing, and the detection of specific IgE (sIgE) antibodies in the serum [4].

The sensitivity and specificity of skin tests and IgE determination are generally difficult to compare due to the use of different methodologies. Even within the established system, variations exist due to the continuous development from RAST to CAP‐FEIA and finally to ImmunoCAP. Similarly, the sensitivity of skin tests is challenging to compare because of the use of different venom preparations on the market and in the varying interpretations of borderline skin test results. Skin test sensitivity varies between 63% and 100%, and specificity between 80% and 97%, depending on concentrations and venom used [5, 6, 7, 8]. In a more recent study, the sensitivity of the intradermal test (IDT) was 91% for vespid venom and 96% for bee venom [9]. Reported sensitivities for sIgE determination to bee venom range from 96% to 97% (specificity: 70%–82%), while for vespid venom, sensitivities are 88%–97% (specificity: 63%–82%) [9, 10]. The basophil activation test (BAT) proved to be a helpful additional tool because of its higher specificity [9] and lower rate of double‐positive results [11]. It has also been demonstrated that the BAT enabled the diagnosis of insect venom allergy in patients with a negative skin test and negative specific IgE [12]. Furthermore, it could be shown that patients with severe sting reactions exhibit a higher CD63 upregulation than patients with mild reactions [13]. However, due to the recurrent presence of non‐responders, the relatively low sensitivity, as well as the high effort, cost, and restriction to specialized centers, the BAT has never been fully established in routine clinical practice.

The introduction of molecular allergy diagnosis (MAD) was considered to significantly improve the diagnosis of insect venom allergy. The initial hope that the sensitivity of the two major allergens, Api m 1 from bee venom and Ves v 5 from vespid venom, would be sufficient for an accurate diagnosis [14] was not confirmed. For the diagnosis of vespid venom allergy, the additional incorporation of Ves v 1 was required to increase sensitivity from 85%–92% to 92%–96% [15, 16]. Several studies have shown that Api m 1 alone, as well as the complete bee venom panel (Api m 1, 2, 3, 5, and 10), fail to achieve sufficient sensitivity for diagnosing bee venom allergy. The overall sensitivity of the available bee venom allergen panel is relatively low at 72% [17], primarily due to the limited sensitivity of Api m 1, which ranges between 57% and 62% [16, 18, 19].

Therefore, MAD is not useful because the sensitivity of the bee venom allergen panel is insufficient.

The diagnostic process is straightforward if a patient is positive for only one venom. However, double positivity has been observed in up to 61.5% of patients [11, 14], sometimes reflecting laboratory cross‐reactivity, but often representing genuine sensitizations to molecular allergens, many of which are not clinically relevant for systemic sting reactions [20]. It has been demonstrated that the presence of IgE can also contribute to large local reactions; individuals with so‐called asymptomatic sensitizations were shown to have a tenfold increased risk of developing such reactions [21]. While the sensitivity of individual diagnostic tests is already high, combining different test modalities can further improve diagnostic accuracy. This study aimed to assess the sensitivity and specificity of currently available tests, both individually and in combination, and to identify the most reliable and simple approach for confirming HVA. Additionally, we attempted to calculate a vespid/bee venom sIgE ratio as a simple method to distinguish between bee and vespid venom allergy, minimizing the risk of overtreatment or incorrect venom selection.

## 2 Methods

### 2.1 Subjects

The study included 1060 subjects divided into three groups: we retrospectively analyzed data from 190 patients with allergic reactions to bee venom and 809 patients with allergic reactions to vespid venom, all of whom had a history of systemic sting reactions. Patients with clinically relevant sensitization to bee and vespid venom were not included in the study. Sixty‐one non‐allergic individuals who had no history of systemic sting reactions or large local reactions served as negative controls for both groups. The study was approved by the ethics committee of the Medical University of Graz (No. 25‐465 ex 12/13), and all patients signed an informed consent.

### 2.2 Determination of sIgE

Total IgE (tIgE) as well as sIgE to bee and vespid venom preparations and all available molecular allergens at this time (rApi m 1, rApi m 2, rApi m 3, rApi m 5, rApi m 10, rVes v 1, and rVes v 5) in the patients' sera were measured using ImmunoCAP 1000 (Thermo Fisher Scientific, Waltham, USA). In general, sIgE values greater than 0.35 kU/L were considered positive, but further analyses using 0.1 kU/L as an additional cut‐off have been performed.

### 2.3 Skin Tests

The nature of sensitization was confirmed by IDT (0.02 mL of 0.01, 0.1, and 1 μg/mL) with aqueous, purified bee and vespid venom preparations (ALK‐Abelló, Hørsholm, Denmark). Intradermal test results were considered positive if a wheal exceeding 5 mm in diameter and concomitant erythema were observed.

Skin prick tests (SPT) have been performed using allergen preparations from ALK‐Abelló in three different concentrations (10, 100, and 300 μg/mL). Test results were considered positive if the wheal was larger than 3 mm in diameter with concomitant erythema.

### 2.4 BAT

All reagents and allergens were from Bühlmann, and BATs were performed according to the manufacturer's instructions (Bühlmann Laboratories AG, Schönenbuch, Switzerland). Cells were stained with anti‐CD63‐FITC and anti‐CCD3‐PE, and samples were analyzed using 3‐color flow cytometry (FC500; Beckman Coulter, Fullerton, USA). Results were considered positive if the CD63 basophil response was ≥ 10%.

### 2.5 Determination of the Relevant Insect

Determination of the relevant insect was based on the clinical history, including specific criteria such as the circumstances of the sting, the season, and the presence of a retained sting. In addition, all patients underwent the full set of diagnostic tests. By considering these findings in combination, the relevant insect was ultimately identified.

### 2.6 Statistical Analyses

Patient characteristics are descriptively summarized using median and range, as well as absolute and relative frequencies. For the analysis of sensitivity and specificity of the routinely performed diagnostic tests (sIgE determination, IDT, SPT, and BAT), patients were included only if results for all diagnostic tests, except the SPT, were available. Sensitivity and specificity for sIgE determinations were calculated using the standard cut‐off of 0.35 kU/L, as well as the additional cut‐off of 0.1 kU/L, irrespective of tIgE levels. Furthermore, in a third analysis, we combined the two cut‐offs as follows: for subjects with tIgE levels ≥ 30 kU/L, the cut‐off for sIgE was set at 0.35 kU/L; otherwise, a cut‐off of 0.1 kU/L was implemented (= dual cut‐off approach). To determine specificity, individuals without large local reactions or systemic sting reactions served as controls.

For the combination of diagnostic tests, those with the highest individual sensitivity were selected as the starting point. From there on, we analyzed how much each additional test further increased sensitivity.

The vespid/bee‐ and bee/vespid‐ sIgE ratios were only assessed for patients with sIgE levels ≥ 0.35 kU/L to either bee venom or vespid venom preparations. The cut‐off was set at the first instant of maximum specificity.

All statistical analyses were performed using R version 4.1.0. The function epi.tests() from the package “epiR” was used to calculate sensitivities and specificities along with exact binomial 95% confidence intervals.

## 3 Results

In total, data of 190 patients with bee venom allergy and 809 patients with vespid venom allergy have been analyzed. Sixty‐one non‐allergic individuals served as controls (Table S1). However, only 112 of the patients allergic to bee venom and 460 of those allergic to vespid venom had complete diagnostic test data and are thus considered further. The demographic data of the included subjects are presented in Table 1. Sensitivity and specificity of the routinely performed diagnostic tests (sIgE determination, IDT, skin prick test (SPT), as well as the basophil activation test) were evaluated.

**TABLE 1:** Demographic data for all three study groups. Only patients with all diagnostic test results were included.

	Bee venom group (*n* = 112)	Vespid venom group (*n* = 460)	Negative control group (*n* = 61)
Age, years	41.5 (16–76)	48 (12–82)	35 (19–61)
Sex			
Male	59 (52.7%)	230 (50.1%)	13 (21.3%)
Female	53 (47.3%)	229 (49.9%)	48 (78.8%)
Grade of systemic reaction according to Ring/Messmer			
Grade I	7 (6.3%)	14 (3.0%)	n.a.
Grade II	78 (69.6%)	303 (65.9%)	n.a.
Grade III	26 (23.2%)	138 (30.0%)	n.a.
Grade IV	1 (0.9%)	5 (1.1%)	n.a.

*Note:* Data are presented as median (range) or as *n* (%).

Abbreviation: n.a., not applicable.

### 3.1 Bee Venom Allergy

Complete diagnostic test results were available for 112 patients.

Using a cut‐off of 0.35 kU/L, the sensitivity of sIgE to bee venom preparation was 85.7%, while for sIgE to all bee venom allergens (positive if at least one of the five bee venom allergens was positive) the sensitivity was 68.8%. The specificity was 86.9% for sIgE to bee venom preparation and 90.2% for sIgE to bee venom allergens (Table 2).

**TABLE 2:** Individual sensitivities and specificities of the different diagnostic tests (using different cut‐offs for positivity) for bee venom allergy.

Test modality	Sensitivity [%] (95% CI)	Specificity [%] (95% CI)
sIgE bee venom preparation (cut‐off 0.35 kU/L)	85.7 (77.8–91.6)	86.9 (75.8–94.2)
sIgE bee venom preparation (cut‐off 0.1 kU/L)	97.3 (92.4–99.4)	72.1 (59.2–82.9)
sIgE bee venom preparation adapted^a^	94.6 (88.7–98.0)	78.7 (66.3–88.1)
sIgE to any molecular allergen (cut‐off 0.35 kU/L)	68.8 (59.3–77.2)	90.2 (79.8–96.3)
sIgE to any molecular allergen (cut‐off 0.1 kU/L)	88.4 (81.0–93.7)	80.3 (68.2–89.4)
IDT (any concentration)	89.3 (82.0–94.3)	96.7 (88.7–99.6)
IDT (only 0.01 or 0.1 μg/mL)	68.8 (59.3–77.2)	98.4 (91.2–100.0)
BAT Buehlmann	73.2 (64.0–81.1)	98.4 (91.2–100.0)

*Note:* 112 patients were used for determination of sensitivity and 61 healthy controls for specificity.

^a^ If tIgE was < 30, a cut‐off of 0.1 kU/L was applied; otherwise, the cut‐off for sIgE was set at 0.35 kU/L.

Lowering the cut‐off to 0.1 kU/L led to an increase in sensitivity (97.3% and 88.4%, respectively); however, specificity decreased to 72.1% and 80.3%, respectively. Using the lower cut‐off of 0.1 kU/L only in patients with tIgE levels below 30 kU/L markedly increased the sensitivity for sIgE to bee venom preparation to 94.6%, while the specificity was slightly lower at 78.7% compared to using the standard cut‐off in all patients (Table 2).

Analyzing all three concentrations of the IDT, specificity was higher (96.7%) compared to the determination of sIgE, with a sensitivity of 89.3%. Excluding results of the highest concentration led to a further increase in specificity (98.4%), but sensitivity decreased to 68.8%. The BAT also achieved a specificity of 98.4%; however, its sensitivity was relatively low at only 73.2% (Table 2).

**FIGURE 1:**
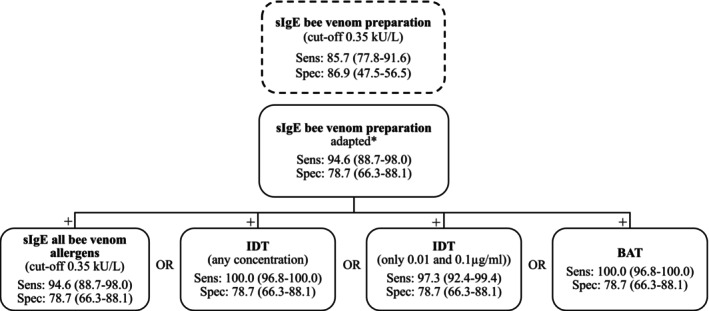
Sensitivities (%) and specificities (%) for combinations of diagnostic tests for bee venom allergy. Sensitivity can be increased to 100% if the sIgE determination to bee venom preparation is either combined with an IDT or a BAT. An additional analysis of the available panel of bee venom components did not increase sensitivity. *If tIgE was < 30, the cut‐off for sIgE was set at 0.1 kU/L, otherwise the usual cut‐off of 0.35 kU/L was used.

A combination of tests enhanced overall sensitivity to 100%, as depicted in Figure 1. Specifically, an adapted analysis was applied that incorporated two cut‐offs for sIgE to bee venom, depending on tIgE levels: if tIgE was < 30 kU/L, a cut‐off of 0.1 kU/L was used; for higher tIgE levels, the cut‐off was set at 0.35 kU/L. Combined with an additional IDT, this approach maximized sensitivity at 100.0%, while specificity was 78.7%. Notably, when the highest concentration of the IDT was excluded from analysis, specificity remained at 78.7%, while sensitivity decreased slightly to 97.3%. Furthermore, employing a BAT as a supplementary test also achieved a sensitivity of 100.0% and a specificity of 78.7%. Molecular allergy diagnosis did not improve sensitivity while specificity again stayed at 78.7%.

Interestingly, incorporating an SPT as a secondary measure showed potential for both, increased sensitivity (100.0%; 95% CI 83.9–100.0) and specificity (77.0%; 95% CI 64.5–86.8). However, this analysis was limited to only 21 patients with bee venom allergy due to the unavailability of SPT results.

### 3.2 Vespid Venom Allergy

For 460 patients with vespid venom allergy, complete diagnostic test results were available.

Using a cut‐off of 0.35 kU/L, the sensitivity for sIgE to vespid venom preparation was 92.2%, while for sIgE to all vespid venom allergens (positive if at least one of the two allergens was positive), sensitivity was 88.5%. The specificity was 80.3% for sIgE to vespid venom preparation and 82.0% for sIgE to vespid venom allergens. Lowering the cut‐off to 0.1 kU/L increased sensitivity to 98.0% and 97.0%, respectively, but reduced specificity to 54.1% and 65.6%, respectively. Lowering the cut‐off to 0.1 kU/L only in patients with tIgE levels below 30 kU/L, the sensitivity for sIgE to vespid venom preparation increased to 97.0%, while specificity was 62.3% (Table 3).

**TABLE 3:** Individual sensitivities and specificities of the different diagnostic tests (using different cut‐offs for positivity) for vespid venom allergy.

Test modality	Sensitivity [%] (95% CI)	Specificity [%] (95% CI)
sIgE vespid venom preparation (cut‐off 0.35 kU/L)	92.2 (89.3–94.5)	80.3 (68.2–89.4)
sIgE vespid venom preparation (cut‐off 0.1 kU/L)	98.0 (96.3–99.1)	54.1 (40.8–66.9)
sIgE vespid venom preparation adapted^a^	97.0 (94.9–98.3)	62.3 (49.0–74.4)
sIgE to any molecular allergen (cut‐off 0.35 kU/L)	88.5 (85.2–91.2)	82.0 (70.0–90.6)
sIgE to any molecular allergen (cut‐off 0.1 kU/L)	97.0 (94.9–98.3)	65.6 (52.3–77.3)
IDT (any concentration)	94.8 (92.3–96.6)	68.9 (55.7–80.1)
IDT (only 0.01 or 0.1 μg/mL)	83.5 (79.8–86.8)	82.0 (70.0–90.6)
BAT Buehlmann	84.3 (80.7–87.5)	75.4 (62.7–85.5)

*Note:* 460 patients were used for determination of sensitivity and 61 healthy controls for specificity.

^a^ If tIgE was < 30 kU/L, a cut‐off of 0.1 kU/L was applied; otherwise, the cut‐off for sIgE was set at 0.35.

When analyzing all three concentrations of the intradermal test, specificity was 68.9%, with a sensitivity of 94.8%. Excluding the results of the highest concentration led to the highest specificity of 82.0%, but sensitivity decreased to 83.5%. The BAT demonstrated a specificity of 75.4% and its sensitivity was among the lowest (84.3%, Table 3).

**FIGURE 2:**
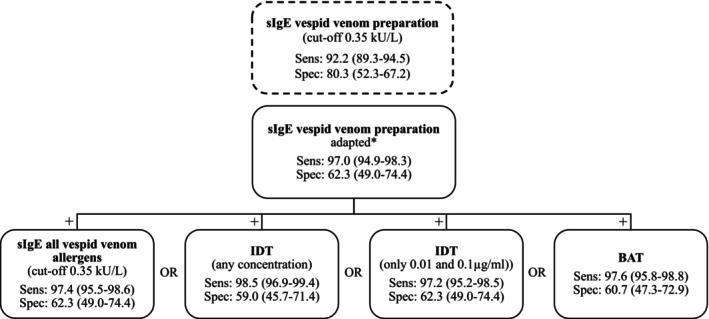
Sensitivities (%) and specificities (%) for combinations of diagnostic tests for vespid venom allergy. The highest sensitivity can be achieved if the determination of sIgE to vespid venom preparation is combined with an IDT (including all three concentrations). *If tIgE was < 30, the cut‐off for sIgE was set at 0.1 kU/L; otherwise the usual cut‐off of 0.35 kU/L was used.

The combination of diagnostic tests led to an improvement in overall sensitivity, though it did not reach 100% (see Figure 2). The highest sensitivity (98.5%) was obtained by combining the dual cut‐off approach for sIgE to vespid venom preparation and an intradermal test (all concentrations), whereas specificity slightly decreased to 59.0%. Excluding the highest skin test concentration improved sensitivity to 97.2% and also increased specificity to 62.3%. Adding a BAT as a second diagnostic test slightly increased sensitivity to 97.6% while slightly reducing specificity to 60.7%. Molecular allergy diagnosis improved sensitivity to 97.4% while specificity stayed at 62.3%.

Performing an additional SPT further improved sensitivity (99.1%; 95% CI 95.3–100.0) and slightly decreased specificity (59.0%; 95% CI 45.7–71.4), though this analysis was limited to 116 patients with vespid venom allergy due to missing SPT results.

### 3.3 Bee & Vespid Venom Allergy

**FIGURE 3:**
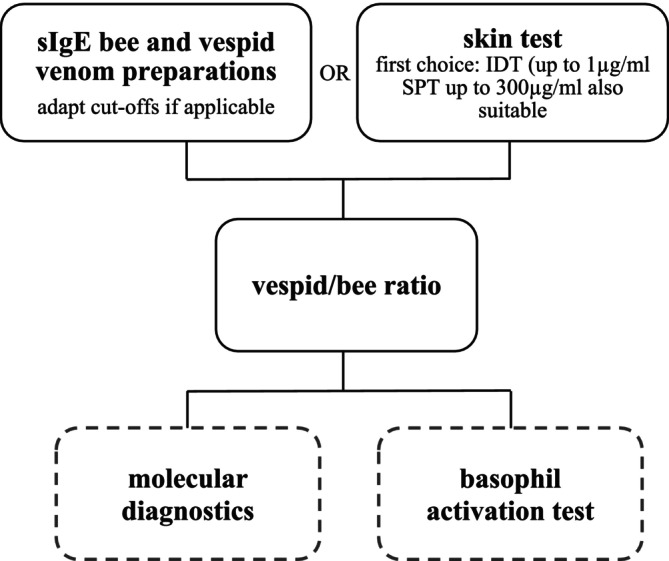
Simple algorithm for diagnostic work‐up. Start with either sIgE or skin testing; if sIgE is available, also determine the vespid/bee ratio. In selected patients, molecular diagnostics or basophil activation testing may be necessary.

To facilitate clinical decision‐making, Figure 3 depicts a simplified diagnostic algorithm for Hymenoptera venom allergy, outlining the main steps of the diagnostic work‐up.

### 3.4 Differentiation Between Bee and Vespid Venom Allergy

For this analysis, only patients with sIgE levels ≥ 0.35 kU/L to either bee venom (*n* = 85) or vespid venom (*n* = 366) preparations were included. If the vespid/bee ratio was ≥ 2.68, the patient was identified as vespid venom allergic with a sensitivity of 59.3% (95% CI 54.1–64.4) and a specificity of 100.0% (95% CI 95.8–100.0).

**FIGURE 4:**
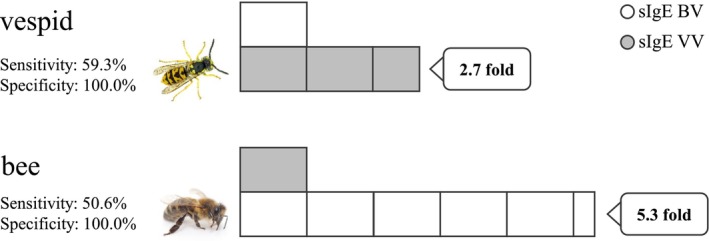
Graphical presentation of sIgE ratios. If sIgE levels to vespid venom are ≥ 2.68 times higher than sIgE levels to bee venom, the patient is identified as vespid venom allergic with a sensitivity of 59.3% and a specificity of 100.0%. A bee/vespid ratio of ≥ 5.34 identifies bee venom‐allergic patients with a sensitivity of 50.6% and a specificity of 100.0%.

Conversely, a bee/vespid ratio of ≥ 5.34 identified bee venom‐allergic patients with a sensitivity of 50.6% (95% CI 39.5–61.6) and a specificity of 100.0% (95% CI 99.0–100.0) (see Figure 4).

Using the proposed ratios, the causative venom was correctly identified in 111/190 (58.4%) bee venom allergic patients as well as 630/809 (77.9%) vespid venom allergic patients in the total study population. Importantly, the ratio is not a stand‐alone solution given its low sensitivity, but rather a practical rule of thumb when tests such as the basophil activation test or molecular diagnostics are unavailable.

## 4 Discussion

The sensitivities of the individual diagnostic tests with the standard cut‐off varied, with bee venom showing a range between 73.2% and 89.3%, while vespid venom ranged between 84.3% and 94.8%.

For both bee and vespid venom, the intradermal test showed the highest sensitivity, with 89.3% for bee venom and 94.8% for vespid venom. Slightly lower sensitivities were observed for specific IgE determination, at 85.7% for bee venom and 92.2% for vespid venom.

The individual routine diagnostic methods already demonstrated very high sensitivity. The adapted approach, which involved lowering the cut‐off to 0.1 kU/L when total IgE levels were below 30 kU/L, resulted in high sensitivities, 94.6% for bee venom and 97.0% for vespid venom, while maintaining an acceptable decrease in specificity at least for bee venom allergic patients.

The combination of conventional tests, such as intradermal testing and sIgE determination, led to a sensitivity of 100.0% for bee venom and 98.5% for vespid venom. Interestingly, the combination of sIgE determination and prick testing also yielded comparably high sensitivities, with 100% (95% CI 83.9–100.0) for bee venom and 99.1% (95% CI 95.3–100.0) for vespid venom. However, it is important to note the lower patient numbers, particularly in the bee venom allergy cohort. However, since the SPT is more cost‐effective and much easier to perform, this combination is certainly an interesting option from both a time and financial perspective. Additional methods, such as the basophil activation test or molecular allergy diagnostics, failed to improve diagnostic performance. Although these tests can be beneficial in specific cases, we did not observe a consistent overall effect.

Increasing sensitivity comes at the expense of specificity. Our goal was to improve sensitivity while avoiding expensive or complex tests. Instead of adding additional diagnostics, we simply lowered the cut‐off for IgE determination to 0.1 kU/L. If this adjustment were applied to all patients, it would significantly reduce specificity. However, limiting the lower cut‐off to patients with a tIgE below 30kU/L may be a reasonable approach, especially to diagnose bee venom allergy, as it only moderately decreases overall specificity. In the case of vespid venom allergy, the specificity of the dual cut‐off approach is considerably lower, which increases the risk of over‐diagnosis and over‐treatment. A general problem is that up to 66.7% of the general population may show sensitization to insect venoms [22, 23], depending on their total IgE. We now know that IgE also plays a role in many cases of enhanced local reactions. Nevertheless, when lowering the cut‐off to 0.1, one must of course expect that asymptomatic sensitizations will also be captured. Therefore, this method should primarily be applied in severe, potentially life‐threatening reactions.

The sIgE determination using the CAP system results in double‐positive findings for bee and vespid venom in 55%–61.5% of patients [11, 14, 24]. Similarly, the intradermal test shows double‐positive results in 47.9% of patients [11]. Molecular allergy diagnostics with the available allergens and the BAT demonstrate a lower rate of double‐positive results in the current study, which has previously been observed [11].

The main issue is the low sensitivity of the bee venom panel, which fails to diagnose over 30% of bee venom allergic patients, naturally leading to fewer double‐positive findings [17]. The BAT also shows fewer double‐positive results but can sometimes be negative in cases of mild reactions [25]. The determination of specific IgE against MUXF, targeting CCD, is, unfortunately, not suitable for distinguishing between a true double sensitization and a CCD‐based cross‐reactivity either. The issue is that patients with genuine polysensitization were also sensitized to MUXF, and in fact, CCD‐based cross‐reactivity confirmed by Western blot immunoassay could only be demonstrated in about half of the cases through MUXF‐specific IgE determination [11].

However, it has been shown that, despite the high rate of double‐positive findings, patients undergoing VIT with only one insect venom were adequately protected. This suggests that many of these double sensitizations are clinically irrelevant [24].

To address this issue, we attempted to calculate a vespid/bee ratio. This ratio is designed for safety: its specificity is 100% if the ratio applies, meaning no patient with a clinically relevant allergy to bee and vespid venom would have been missed in this study. However, the ratio's low sensitivity suggests that many more patients may have clinically irrelevant double‐positive findings. Importantly, this does not represent a stand‐alone solution due to its low sensitivity. Rather, it is intended as a practical rule of thumb in situations where other tests, such as the BAT or MAD, are not available. Interestingly, another study group independently calculated a similar ratio for diagnosing bee venom allergy and reached almost identical results, supporting the robustness of the ratio [26]. Unfortunately, they did not publish a ratio for vespid venom.

In summary, the sensitivity of established tests is already very high and can be further increased by adapting the cut‐off in cases of low total IgE and by combining tests. Therefore, we would recommend starting with either the determination of sIgE to bee and vespid venom preparations or skin testing. If sIgE is available, the vespid/bee ratio should be determined, since this simple method allows for the quick exclusion of irrelevant double sensitizations without the need for additional expensive tests. However, in some patients, additional tests such as molecular diagnostics or the BAT may still provide valuable information.

## Author Contributions

L.A.‐G. and G.J.S.: conceptualization. G.J.S.: supervision. L.A.‐G., E.S., U.Č., E.J., K.L., B.B., G.J.S.: investigation. G.P., S.A.H., L.A.‐G.: formal analysis. E.S., U.Č., E.J., G.P., S.A.H., K.L.: methodology. L.A.‐G., E.S., G.J.S.: writing‐original draft. L.A.‐G., E.S., U.Č., G.P., S.A.H., E.J., K.L., B.B., G.J.S.: writing‐review and editing. The corresponding author attests that all listed authors meet authorship criteria and that no others meeting the criteria have been omitted.

## Conflicts of Interest

Dr. Sturm reports grants from ALK‐Abelló, personal fees from ALK‐Abelló, personal fees from Allergopharma, personal fees from Novartis, and personal fees from Stallergenes‐Greer, outside the submitted work. All other authors have no conflicts of interest to declare.

## Supporting information


**Table S1:** (a) Demographic data and diagnostic test results for bee venom for all three study groups. (b) Diagnostic test results for vespid venom for all three study groups. (c) Diagnostic test results for bee and vespid venom (= positive results for bee and vespid venom) for all three study groups.

## Data Availability

The data that support the findings of this study are available from the corresponding author upon reasonable request.
