# Supplementary material for: A Straightforward Algorithm for Diagnosing Hymenoptera Venom Allergy and Identifying the Relevant Venom for Immunotherapy
Source: Allergy. 2025 Nov 12;81(6):2103–10. doi: 10.1111/all.70154 (PMC13256283; doi:10.1111/all.70154)
Supplement: Supplementary file 1 — Table S1: (a) Demographic data and diagnostic test results for bee venom for all three study groups. (b) Diagnostic test results for vespid venom for all three study groups. (c) Diagnostic test results for bee and vespid venom (= positive results for bee and vespid venom) for all three study groups. [file ALL-81-2103-s001.docx]

| Supplementary table 1a: Demographic data and diagnostic test results for **bee venom** for all three study groups. | | | | | |
| --- | --- | --- | --- | --- | --- |
| **Characteristic** | **overall**  N = 1060^1^ | **Bee venom group**  N = 190^1^ | **Vespid venom group**  N = 809^1^ | **Negative control group**  N = 61^1^ | |
| age [years], range | 46 (33, 56) | 40 (29, 54) | 48 (35, 58) | 35 (28, 50) | |
| gender |  |  |  |  | |
| male | 499 (47%) | 104 (55%) | 382 (47%) | 13 (21%) | |
| female | 559 (53%) | 86 (45%) | 425 (53%) | 48 (79%) | |
| Unknown | 2 | 0 | 2 | 0 | |
| sIgE bee venom preparation (cut-off 0.35 kU/L) |  |  |  |  | |
| neg. | 517 (49%) | 25 (13%) | 439 (54%) | 53 (87%) | |
| pos. | 543 (51%) | 165 (87%) | 370 (46%) | 8 (13%) | |
| sIgE bee venom preparation (cut-off 0.10 kU/L) |  |  |  |  | |
| neg. | 276 (26%) | 4 (2.1%) | 228 (28%) | 44 (72%) | |
| pos. | 784 (74%) | 186 (98%) | 581 (72%) | 17 (28%) | |
| sIgE bee venom preparation adapted* |  |  |  |  | |
| neg. | 444 (42%) | 11 (5.8%) | 385 (48%) | 48 (79%) | |
| pos. | 613 (58%) | 179 (94%) | 421 (52%) | 13 (21%) | |
| Unknown | 3 | 0 | 3 | 0 | |
| sIgE to any molecular allergen (bee, cut-off 0.35 kU/L) |  |  |  |  | |
| neg. | 416 (53%) | 52 (28%) | 309 (58%) | 55 (90%) | |
| pos. | 362 (47%) | 131 (72%) | 225 (42%) | 6 (9.8%) | |
| Unknown | 282 | 7 | 275 | 0 | |
| sIgE to any molecular allergen (bee, cut-off 0.10 kU/L) |  |  |  |  | |
| neg. | 273 (35%) | 16 (8.7%) | 208 (39%) | 49 (80%) | |
| pos. | 505 (65%) | 167 (91%) | 326 (61%) | 12 (20%) | |
| Unknown | 282 | 7 | 275 | 0 | |
| IDT bee venom (any concentration) |  |  |  |  | |
| neg. | 630 (62%) | 17 (9.4%) | 554 (71%) | 59 (97%) | |
| pos. | 393 (38%) | 164 (91%) | 227 (29%) | 2 (3.3%) | |
| Unknown | 37 | 9 | 28 | 0 | |
| IDT bee venom (only 0.01 or 0.1 µg/ml) |  |  |  |  | |
| neg. | 829 (81%) | 58 (32%) | 711 (91%) | 60 (98%) | |
| pos. | 194 (19%) | 123 (68%) | 70 (9.0%) | 1 (1.6%) | |
| Unknown | 37 | 9 | 28 | 0 | |
| BAT Buehlmann bee venom |  |  |  |  | |
| neg. | 534 (82%) | 32 (27%) | 442 (94%) | 60 (98%) | |
| pos. | 116 (18%) | 88 (73%) | 27 (5.8%) | 1 (1.6%) | |
| Unknown | 410 | 70 | 340 | 0 | |
| SPT bee venom |  |  |  |  | |
| neg. | 194 (80%) | 8 (27%) | 128 (84%) | 58 (95%) | |
| pos. | 50 (20%) | 22 (73%) | 25 (16%) | 3 (4.9%) | |
| Unknown | 816 | 160 | 656 | 0 | |
| ^1^ Median (Q1, Q3); n (%) | | | | |  |
| * If tIgE was <30, a cut-off of 0.1 kU/L was applied; otherwise, the cut-off for sIgE was set at 0.35 kU/L. | | | | |  |
| IDT - intradermal test; SPT - skin prick test | | | | |  |

| Supplementary table 1b: Diagnostic test results for **vespid venom** for all three study groups. | | | | | |
| --- | --- | --- | --- | --- | --- |
| **Characteristic** | **Overall**  N = 1060^1^ | **Bee venom group**  N = 190^1^ | **Vespid venom group**  N = 809^1^ | **Negative control group**  N = 61^1^ | |
| sIgE vespid venom preparation (cut-off 0.35 kU/L) |  |  |  |  | |
| neg. | 199 (19%) | 103 (54%) | 47 (5.8%) | 49 (80%) | |
| pos. | 860 (81%) | 86 (46%) | 762 (94%) | 12 (20%) | |
| Unknown | 1 | 1 | 0 | 0 | |
| sIgE vespid venom preparation (cut-off 0.1 kU/L) |  |  |  |  | |
| neg. | 92 (8.7%) | 49 (26%) | 10 (1.2%) | 33 (54%) | |
| pos. | 967 (91%) | 140 (74%) | 799 (99%) | 28 (46%) | |
| Unknown | 1 | 1 | 0 | 0 | |
| sIgE vespid venom preparation adapted* |  |  |  |  | |
| neg. | 140 (13%) | 85 (45%) | 17 (2.1%) | 38 (62%) | |
| pos. | 916 (87%) | 104 (55%) | 789 (98%) | 23 (38%) | |
| Unknown | 4 | 1 | 3 | 0 | |
| sIgE to any molecular allergen (vespid, cut-off 0.35 kU/L) |  |  |  |  | |
| neg. | 266 (25%) | 144 (76%) | 72 (8.9%) | 50 (82%) | |
| pos. | 793 (75%) | 45 (24%) | 737 (91%) | 11 (18%) | |
| Unknown | 1 | 1 | 0 | 0 | |
| sIgE to any molecular allergen (vespid, cut-off 0.35 kU/L) |  |  |  |  | |
| neg. | 144 (14%) | 90 (48%) | 14 (1.7%) | 40 (66%) | |
| pos. | 915 (86%) | 99 (52%) | 795 (98%) | 21 (34%) | |
| Unknown | 1 | 1 | 0 | 0 | |
| IDT vespid venom (any concentration) |  |  |  |  | |
| neg. | 166 (16%) | 95 (52%) | 29 (3.7%) | 42 (69%) | |
| pos. | 857 (84%) | 86 (48%) | 752 (96%) | 19 (31%) | |
| Unknown | 37 | 9 | 28 | 0 | |
| IDT vespid venom (only 0.01 or 0.1 µg/ml) |  |  |  |  | |
| neg. | 351 (34%) | 148 (82%) | 153 (20%) | 50 (82%) | |
| pos. | 672 (66%) | 33 (18%) | 628 (80%) | 11 (18%) | |
| Unknown | 37 | 9 | 28 | 0 | |
| BAT Buehlmann vespid venom |  |  |  |  | |
| neg. | 224 (35%) | 105 (88%) | 73 (16%) | 46 (75%) | |
| pos. | 425 (65%) | 15 (13%) | 395 (84%) | 15 (25%) | |
| Unknown | 411 | 70 | 341 | 0 | |
| SPT vespid venom |  |  |  |  | |
| neg. | 92 (38%) | 22 (73%) | 22 (14%) | 48 (79%) | |
| pos. | 152 (62%) | 8 (27%) | 131 (86%) | 13 (21%) | |
| Unknown | 816 | 160 | 656 | 0 | |
| ^1^ Median (Q1, Q3); n (%) | | | | |  |
| * If tIgE was <30, a cut-off of 0.1 kU/L was applied; otherwise, the cut-off for sIgE was set at 0.35 kU/L. | | | | |  |
| IDT - intradermal test; SPT - skin prick test | | | | |  |

| Supplementary table 1c: Diagnostic test results for bee and vespid venom (=positive results for bee and vespid venom) for all three study groups. | | | | | |
| --- | --- | --- | --- | --- | --- |
| **Characteristic** | **Overall**  N = 1060^1^ | **Bee venom group**  N = 190^1^ | **Vespid venom group**  N = 809^1^ | **Negative control group**  N = 61^1^ | |
| sIgE bee/vespid venom preparation (cut-off 0.35 kU/L) |  |  |  |  | |
| neg. | 606 (57%) | 104 (55%) | 445 (55%) | 57 (93%) | |
| pos. | 453 (43%) | 85 (45%) | 364 (45%) | 4 (6.6%) | |
| Unknown | 1 | 1 | 0 | 0 | |
| sIgE bee/vespid venom preparation (cut-off 0.1 kU/L) |  |  |  |  | |
| neg. | 327 (31%) | 50 (26%) | 229 (28%) | 48 (79%) | |
| pos. | 732 (69%) | 139 (74%) | 580 (72%) | 13 (21%) | |
| Unknown | 1 | 1 | 0 | 0 | |
| sIgE bee/vespid venom preparation adapted* |  |  |  |  | |
| neg. | 527 (50%) | 86 (46%) | 390 (48%) | 51 (84%) | |
| pos. | 529 (50%) | 103 (54%) | 416 (52%) | 10 (16%) | |
| Unknown | 4 | 1 | 3 | 0 | |
| sIgE to any molecular allergen (cut-off 0.35 kU/L) |  |  |  |  | |
| neg. | 517 (67%) | 140 (77%) | 320 (60%) | 57 (93%) | |
| pos. | 260 (33%) | 42 (23%) | 214 (40%) | 4 (6.6%) | |
| Unknown | 283 | 8 | 275 | 0 | |
| sIgE to any molecular allergen (cut-off 0.1 kU/L) |  |  |  |  | |
| neg. | 353 (45%) | 89 (49%) | 210 (39%) | 54 (89%) | |
| pos. | 424 (55%) | 93 (51%) | 324 (61%) | 7 (11%) | |
| Unknown | 283 | 8 | 275 | 0 | |
| IDT bee/vespid venom (any concentration) |  |  |  |  | |
| neg. | 713 (70%) | 98 (54%) | 555 (71%) | 60 (98%) | |
| pos. | 310 (30%) | 83 (46%) | 226 (29%) | 1 (1.6%) | |
| Unknown | 37 | 9 | 28 | 0 | |
| IDT bee/vespid venom (only 0.01 or 0.1 µg/ml) |  |  |  |  | |
| neg. | 923 (90%) | 149 (82%) | 714 (91%) | 60 (98%) | |
| pos. | 100 (9.8%) | 32 (18%) | 67 (8.6%) | 1 (1.6%) | |
| Unknown | 37 | 9 | 28 | 0 | |
| BAT Buehlmann bee/vespid venom |  |  |  |  | |
| neg. | 609 (94%) | 107 (89%) | 441 (94%) | 61 (100%) | |
| pos. | 40 (6.2%) | 13 (11%) | 27 (5.8%) | 0 (0%) | |
| Unknown | 411 | 70 | 341 | 0 | |
| SPT bee/vespid venom |  |  |  |  | |
| neg. | 211 (86%) | 23 (77%) | 128 (84%) | 60 (98%) | |
| pos. | 33 (14%) | 7 (23%) | 25 (16%) | 1 (1.6%) | |
| Unknown | 816 | 160 | 656 | 0 | |
| ^1^ Median (Q1, Q3); n (%) | | | | |  |
| * If tIgE was <30, a cut-off of 0.1 kU/L was applied; otherwise, the cut-off for sIgE was set at 0.35 kU/L. | | | | |  |
| IDT - intradermal test; SPT - skin prick test | | | | |  |
